# Medical Management to Treat Chronic Non-healing Ulcers: A Case Series

**DOI:** 10.7759/cureus.51449

**Published:** 2024-01-01

**Authors:** Joseph Jose, Bhanushree Soni, Saji Jose, Jose K Kokkatt

**Affiliations:** 1 Cardiovascular Medicine, Cardiff Health Centre, Manarcad, IND; 2 Preventive Medicine, Jawaharlal Institute of Postgraduate Medical Education and Research, Puducherry, IND; 3 Radiology, Cardiff Health Centre, Manarcad, IND

**Keywords:** chronic non-healing ulcers, autoimmune ulcers, wound care management, immunotherapy, chronic venous ulcers, atypical ulcers

## Abstract

Introduction: Chronic non-healing leg ulcers are skin defects below the knee that resist healing for more than six weeks. They cause physical, emotional, and economic burdens to patients and society.

Objectives: To introduce an innovative medical strategy that targets the chronic inflammation component in non-healing ulcers (NHUs) with rheumatic features and to evaluate its potential effectiveness in achieving complete healing.

Methods: We employed an empirical medical therapy regimen, which combined medications like deflazacort, colchicine, dapsone, hydroxychloroquine, and azathioprine. We retrospectively selected 25 patients with chronic pedal ulcers who underwent our therapy.

Results: The mean duration of ulcers was 7.84 years, and the time to heal was 5.97 months. Among 25 patients, 19 had atypical ulcers, four had venous ulcers, and two had diabetic neuropathy ulcers. Four patients with venous ulcers additionally underwent endovenous laser ablation.

Conclusion: Our medical strategy showed promising results in healing chronic NHUs with rheumatic features without significant steroid-induced adverse effects.

## Introduction

Chronic non-healing leg ulcers pose a significant healthcare challenge globally, characterized by persistent skin defects below the knee level that resist healing for more than six weeks, often extending beyond three months [1,2]. These ulcers can arise from diverse factors such as venous or arterial insufficiency or a combination of both, diabetes, pressure, infections, skin malignancies, and autoimmune disorders [1,3]. Their deleterious effects include pain, infection, compromised mobility, and reduced quality of life. Prevalence rates are notable, affecting around 1% of the adult population and 3.6% of individuals over 65 years old, with even higher incidence observed in specific regions [4].

In India, the occurrence of chronic wounds is estimated at 4.5 per 1000 population [5]. The ramifications of chronic non-healing leg ulcers are profound, imposing a substantial socioeconomic burden due to frequent dressing changes, hospitalizations, and, in extreme cases, amputations, which ultimately lead to poor quality of life and mental health. The etiology and management of these ulcers are intricate, involving a complex interplay of factors like venous insufficiency, arterial diseases, neuropathy, and a range of associated influences such as age, obesity, immobility, smoking, and thrombosis [2]. Approximately 80% of these ulcers are vascular, while about 20-23% have complex causes like vasculitis, pyoderma gangrenosum, and other autoimmune conditions. Thus, most non-vascular ulcers can be considered autoimmune in nature [6].

Conventional treatment modalities encompass compression therapy, wound debridement, topical agents, skin grafts, and vascular interventions [4]. Nonetheless, these approaches are often met with limited efficacy, high costs, or potential complications [7]. A pivotal aspect that warrants heightened attention in the context of chronic non-healing ulcers (NHUs) is the role of extensive inflammation in derailing the normal wound-healing process [8]. This case series introduces an innovative medical strategy that sheds light on its potential effectiveness in achieving complete healing for patients with NHUs.

## Materials and methods

Study setting

The research was conducted at an outpatient hospital center located in Manarcad, Kerala. This center specializes in evaluating patients with varicose veins for chronic NHUs. On a daily basis, the center received approximately two to five patients with pedal ulcers.

Consent and patient recruitment

For the study, 25 individuals above 18 years who had a history of healed chronic pedal ulcers persisting for a duration of six months or more were retrospectively selected as participants after informed consent. Individuals with peripheral arterial insufficiency or co-existing medical conditions such as heart failure, chronic liver disease, chronic renal disease, and chronic infectious diseases like tuberculosis (TB), HIV, hepatitis B, and hepatitis C were excluded from participation. Among the participants in the study, 19 had atypical ulcers, four had venous ulcers, and two had diabetic neuropathic ulcers.

Patient history and investigations

All enrolled patients underwent a comprehensive assessment that included detailed medical history, clinical examination, and ultrasound Doppler scans to screen for arterial and venous insufficiency. An echocardiography and ECG were conducted to rule out heart failure and ischemic heart diseases. Liver function tests (alanine aminotransferase, aspartate aminotransferase, alkaline phosphatase, gamma-glutamyl transferase, and albumin/globulin ratio) and kidney function tests (serum creatinine, urea, and urine microalbumin) were performed to rule out renal and hepatic dysfunction. Additionally, blood tests were conducted, including tests for erythrocyte sedimentation rate (ESR) as an inflammatory marker, complete blood count, HIV, hepatitis B surface antigen, hepatitis C virus (HCV), acid-fast bacilli (AFB) in sputum to rule out TB, and wound cultures to look for wound infections. A wound biopsy and a detailed immunologic assay were not conducted owing to a variety of factors like the patient’s financial constraints, fear of further wound healing delay, and lack of specificity in a definitive diagnosis. However, two of the patients had previously undergone biopsies, which showed vasculitis.

Treatment

The treatment regimen involved administering low-dose oral deflazacort (12 to 30 mg per day) and colchicine (0.5 mg twice a day) to all patients. Adjunct medications such as hydroxychloroquine (HCQ) 200 mg, dapsone 100 mg, and azathioprine 75 mg were administered based on the clinical severity of the wound and added features like polyarthralgia. Gabapentin 300 mg, pregabalin 75 mg, and nortriptyline 50 mg were added for patients with peripheral neuropathy. In case of concomitant wound infection or cellulitis at the time of presentation, an empirical therapy of amoxicillin 500 mg plus clavulanate 125 mg twice daily and injection of amikacin 500 mg was given, and if no response was seen, an appropriate antibiotic based on culture sensitivity was given. Vacuum-assisted debridement or mechanical debridement was employed to get rid of wound slough. All patients were trained for daily self-dressing with topical antibiotics. The patients were reviewed twice weekly and later monthly after sufficient improvement. The wound improvement was assessed clinically based on improvement in wound size, undermining edges, and improvement in symptoms like edema, pain, and itching. Photographs were taken as objective evidence whenever the respective patient provided consent. Upon complete healing, deflazacort was tapered over a two to four-week period based on dosage, and adjunct drugs like colchicine, dapsone, HCQ, or azathioprine were discontinued.

For the patients with venous ulcers diagnosed with significant saphenofemoral or saphenopopliteal incompetence, endovenous laser ablation (EVLA) was performed. All four of our patients with venous ulcers met the previously mentioned criteria and hence all four of them underwent EVLA.

## Results

The age of the participants ranged from 37 to 83 years with a mean (SD) of 59.80 (9.63) years; 12 (48%) of them were males and 13 (52%) were females. The median (interquartile range) duration of ulcers was four (0.68-13) years and the time to heal was five (1.75-6) months. Out of 25 patients, 19 had atypical ulcers, four had venous ulcers, and two had diabetic neuropathy ulcers. Most of the patients had signs of peripheral neuropathy, no family history of ulcers, abnormal ESR, and ulcers of unknown origin. Half of the patients had infected ulcers, eight (32%) of them had skin changes, and eight (32%) had associated diabetes (Table 1). The individual characteristics and treatment regimen details are added in the Supplementary Table in the Appendix.

**Table 1:** Sociodemographic and clinical characteristics of participants recruited from the medical center in 2022 (n = 25). ^*^ Median (interquartile range); ^$^ non-exclusive.

Variable		Mean	SD
Age(in years)		59.80	9.63
BMI		25.49	4.12
Duration of ulcers (in years)*		4	0.68-13
Time for ulcer healing (in months)*		5	1.75-6
-		Frequency (n)	Percentage (%)
Gender	Male	12	48
	Female	13	52
Pain	Moderate	16	64
	Severe	9	36
Edema	Unilateral	14	56
	Bilateral	11	44
Infected	Yes	12	48
	No	13	52
Nerve involvement	Yes	20	80
	No	5	20
Family history of ulcers	Yes	3	12
	No	22	88
Erythrocyte sedimentation rate	Abnormal	20	80
	Normal	5	20
Site of ulcer*	Medial malleolus	10	38.46
	Dorsum of foot	10	38.46
	Lateral malleolus	4	15.38
	Plantar surface of foot	2	7.69
	Lower half of leg	2	7.69
Symptoms	Skin changes	8	32
	Joint stiffness	6	24
	Pruritis	7	32
	None	4	16
Type of ulcer	Venous	4	16
	Neuropathic	2	8
	Atypical	19	76
Co-morbidity^$^	Diabetes mellitus	8	32
	Hypertension	6	24
	Reactive airway disease	1	4
	Stroke	1	4

Case 1

A 72-year-old female with a nine-month-old NHU on the right lower limb presented severe pain and sleep disturbance. She had a history of pedal edema and lower limb pain for 29 years and bilateral numbness in her feet for three years. She has a history of bilateral knee replacement surgery. Previous treatments included antibiotics and metronidazole dressings. But she had no response.

Clinical examination, venous Doppler, and arterial lower limb Doppler showed normal results. Blood work revealed an elevated ESR of 94 mm/hour and a WBC count of 14,000, while other parameters were normal, leading to differential diagnoses of infected atypical ulcers.

The patient was administered deflazacort 30 mg once daily, colchicine 0.5 mg once daily, dapsone 100 mg once daily, and folate 5 mg. A seven-day course of amoxicillin-clavulanic acid (625 mg thrice daily) was prescribed for infection. Following the four-week response, deflazacort was tapered to 6 mg twice daily. Chitosan dressing and vacuum debridement were utilized. Regular follow-up visits occurred every 14 to 28 days, with monitoring of liver function tests, renal function tests, complete blood count, and blood sugar levels monthly.

**Figure 1:**
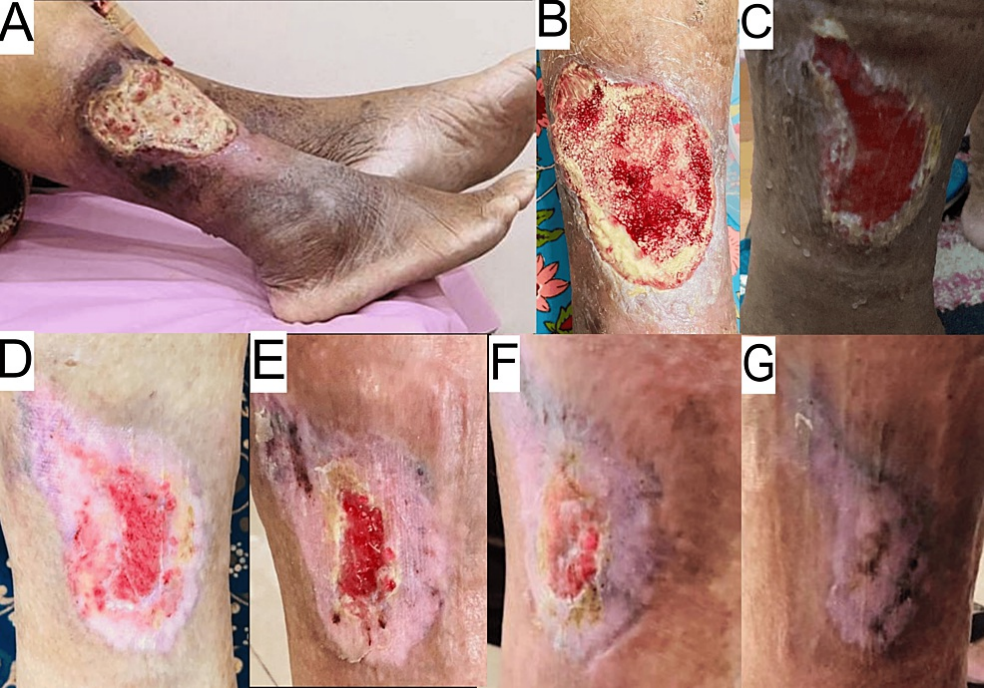
Wound healing progression of case 1. A - 0 weeks, B - two weeks, C - four weeks, D - eight weeks, E - 16 weeks, F - 18 weeks, and G - 20 weeks.

Within two weeks of therapy, the patient reported pain relief, reduced edema, complete ulcer healing, and decreased feet numbness. Complete wound healing was achieved in five months along with restoration of skin color. Glycemic control was maintained throughout the course of therapy, and the ESR decreased to 8 mm/hour. Figure 1 shows wound healing progression.

Case 2

A 63-year-old male with the habit of smoking presented with NHUs on his right foot persisting for 32 years, causing severe pain, pedal edema, and occasional numbness in his foot. Despite previous treatments, no improvement had been observed. No significant medical history or family history of similar issues was reported.

Two chronic ulcers were identified, one on the dorsum (14 x 10 cm) and another on the medial malleolus (10 x 10 cm) with well-defined margins and a sloughy base. A neurological examination revealed a reduced sensation in the right foot. Investigations showed normal arterial and venous flow but elevated ESR.

**Figure 2:**
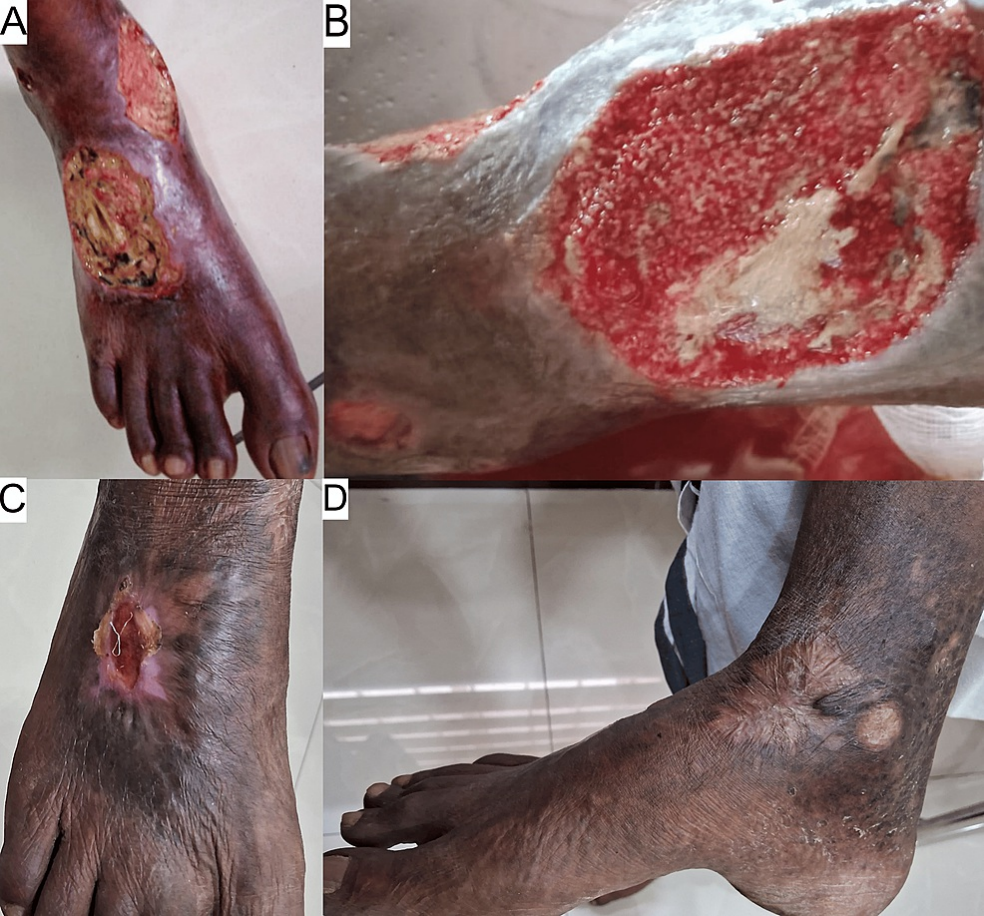
Wound healing progression of case 2. A - 0 weeks, B - one week post vacuum-assisted debridement, and C-D - fully healed.

Presumed to have vasculitis ulcers, treatment included deflazacort (30 mg once daily), colchicine (0.5 mg twice daily), gabapentin, nortriptyline (400 mg twice daily), and topical antibiotic ointment. Injection of amikacin 500 mg for five days and a seven-day course of amoxicillin-clavulanic acid (625 mg twice daily) was prescribed for infection and vacuum-assisted debridement was done. After 12 months, both ulcers healed completely. Medications were gradually tapered and stopped over four weeks post healing, and the patient was advised on smoking cessation. Figure 2 shows the wound healing process of case 2.

Case 3

A 54-year-old diabetic male with bilateral loss of sensation over the plantar surface of his feet presented with NHUs on the right foot persisting for a year. Two ulcers near the 2nd and 3rd metatarsophalangeal joint were observed with a sloughy base and pus. Elevated ESR (64 mm/hour) and high random blood sugar (RBS, 280 mg/dL) were noted. The diagnosis was a neuropathic ulcer with a possible autoimmune component.

**Figure 3:**
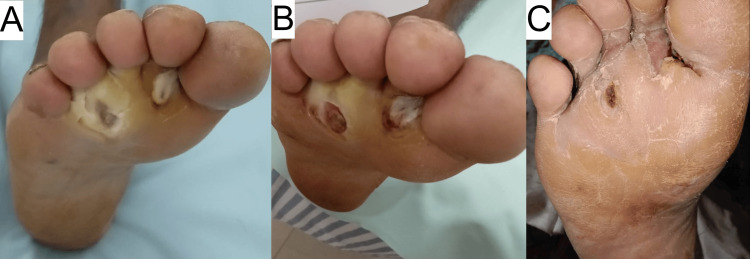
Wound healing progression in case 3. A - 0 weeks, B - eight weeks, C - 16 weeks.

Treatment included colchicine 0.5 mg twice daily, deflazacort 6 mg twice daily, injection of amikacin 500 mg daily (for five days), dapsone 100 mg once daily, gabapentin, nortriptyline once daily, and topical application of metronidazole antibiotic ointment. Vacuum debridement led to rapid pain, edema improvement, and granulation tissue formation. After one month, both ulcers completely healed, with RBS = 154 mg/dL at the time of healing. Dapsone, colchicine, and deflazacort were stopped, while gabapentin and nortriptyline were continued for neuropathic pain management. The patient was advised on glycemic control and foot hygiene. Figure 3 shows the progression of wound healing in case 3.

## Discussion

Chronic NHUs pose significant medical and economic challenges, often leading to severe consequences such as amputations and premature death. Even though the common etiology behind such ulcers is vascular, autoimmune conditions like vasculitis and pyoderma gangrenosum are the most common causes of non-vascular ulcers.

In our study, we explored a novel cheaper treatment approach for chronic NHUs, involving 25 patients with various ulcer types, including venous, diabetic neuropathic, and atypical ulcers, all exhibiting autoimmune components. We implemented an empirical medical therapy regimen, which combined medications deflazacort, colchicine, dapsone, hydroxychloroquine, and azathioprine. This approach resulted in complete wound healing for all patients, with an average healing time of five months. Rapid pain relief, reduced edema, and reduction in rheumatic symptoms like polyarthralgia occurred within one to two weeks of initiating treatment. Few patients also reported improvements in peripheral neuropathy symptoms. Throughout the treatment, we closely monitored inflammatory markers, such as ESR, and glycemic control through RBS measurements.

The existing literature lacks standardized treatment guidelines for chronic NHUs relying mainly on case studies and series [9]. While some studies have explored therapies like autologous platelet-rich plasma, a comparative study concluded that their effect on wound area is non-significant [10]. In the context of autoimmune ulcers such as pyoderma gangrenosum, corticosteroids are a prominent treatment option, sometimes supplemented with colchicine, dapsone, or azathioprine. However, complete wound healing is not consistently achieved, and some cases report mortality [11,12]. Notably, the diagnostic approach for pyoderma gangrenosum and atypical autoimmune ulcers relies on exclusion, but standardized treatment remains elusive.

Despite chronic inflammation being a hallmark factor delaying wound healing, its reversal therapeutically is often overlooked. Chronic NHUs stagnate usually at the early inflammatory stage of wound healing exhibiting overabundant neutrophil infiltration [13,14].

Regarding chronic venous ulcers, some studies suggest potential improvement with adalimumab for ulcers larger than five cm^2^ persisting for at least six months, indicating the possibility of targeted immune suppression in chronic ulcer treatment. However, complete wound resolution was not consistently reported. Although chronic inflammation is believed to play a role in diabetic neuropathic ulcers, there is limited evidence supporting the use of immunosuppressive medications for such ulcers, despite the established utility of steroid therapy in managing peripheral neuropathy.

Our research hypothesis centered on the idea that many chronic NHUs may involve underlying autoimmune or inflammatory factors hindering normal wound healing. Many of our patients presented with associated rheumatic features like polyarthralgia and peripheral neuropathy, which are seen in vasculitis. Additionally, chronic inflammation is implicated not only in non-vascular ulcers but also in chronic venous ulcers and diabetic neuropathy.

To address these underlying mechanisms, we designed an empirical medical therapy approach. Our primary choice of steroid was deflazacort due to its comparable anti-rheumatic properties and a more favorable side-effect profile than prednisolone, particularly concerning hyperglycemia and osteoporosis [15]. We augmented this with colchicine, known for its anti-inflammatory and immunosuppressive effects in vasculitis [16]. Colchicine also demonstrated anti-glycemic actions in patients with gouty arthritis [17], hence potentially playing a key role in preventing steroid-induced hyperglycemia. Notably, our combination therapy of deflazacort and colchicine, although lacking extensive literary evidence, is backed by therapeutic expertise.

In addition to these medications, we introduced dapsone and hydroxychloroquine as adjuncts, given their well-established immunosuppressive effects in vasculitis and pyoderma gangrenosum [11,18].

Furthermore, we expanded our treatment strategy to four patients who presented with severe venous insufficiency, indicated by junctional incompetence grade 4 or higher observed in color Doppler imaging. To address both the venous insufficiency and the chronic inflammation implicated in venous ulcer pathogenesis, we employed a combination of empirical medical therapy and endovascular laser ablation. This newer combined approach aimed to correct venous reflux and enhance venous drainage.

Glucocorticoids are, by far, the most effective anti-inflammatory drugs for treating chronic inflammatory diseases, allergies, and autoimmune pathologies, such as rheumatoid arthritis (RA), asthma, multiple sclerosis, and systemic lupus erythematosus [19,20]. The main concerns for prolonged immunosuppressant therapy with long-term steroids in NHUs would be the risk of infection and delayed wound healing but there are studies negating this concern [6]. Studies show that while glucocorticoids may predispose to skin fragility and thus serve as a risk factor for ulceration in patients with RA, more aggressive therapy for the underlying RA, using disease-modifying antirheumatic drugs (DMARD) and biologic agents such as tumor necrosis factor-alpha inhibitors and rituximab is beneficial [6]. In a large series investigating postoperative wound dehiscence in surgical patients, the use of steroids or immunosuppressive drugs in the 100 days prior to the index surgery was not a risk factor for postoperative wound dehiscence suggesting that steroid therapy may not be a direct risk factor for wound development [6]. Another risk factor to be considered in long-term steroid use would be metabolic side effects. A trial conducted utilizing metformin and systemic glucocorticoid combination therapy showed maintained glycemic control and reduced incidence of metabolic side effects [21]. Since colchicine has demonstrated anti-glycemic effects, it was used to prevent possible metabolic side effects with deflazacort, which has a lower incidence of bone and carbohydrate metabolism side effects compared to commonly used glucocorticoids like prednisolone [15]. To our knowledge, this is a novel treatment approach using low-dose deflazacort and colchicine therapy for immunosuppression. As drugs, glucocorticoids are generally considered immunomodulatory rather than simply immunosuppressive, because of their complex effects on the cells of the immune system. Nevertheless, some pro-inflammatory effects of glucocorticoids have been reported, such as the induction of the expression of NLRP3, a central component of the inflammasome [22].

Limitations

Even though our patients were evaluated for arterial and venous insufficiency, which are the most common etiologies for chronic NHUs, a definitive diagnosis for atypical ulcers through biopsy was not attempted, which would have further aided in proper diagnosis and management. Rather, such patients were labeled to have autoimmune atypical ulcers based on treatment response to immunosuppressants. Hence, other rare causes for chronic ulcers like malignancy and rare chronic infections could not be ruled out in a standard evidence-based manner. Another limitation of the study is the lack of screening for other side effects of long-term immunosuppression due to steroids like osteoporosis, the possibility of acute infections like cellulitis, and loss of muscle mass. However, such side effects were not clinically encountered in our study.

## Conclusions

The management of chronic NHUs typically revolves around addressing their underlying causes or etiology. Conventional approaches involve essential wound care practices, including regular saline dressings, prophylactic antibiotics to prevent infection, compression dressings, and debridement of necrotic tissue. More advanced therapeutic options encompass hyperbaric oxygen therapy, skin grafting, and vacuum-assisted wound debridement. Additionally, for specific types of ulcers, such as venous ulcers, treatment options may include EVLA, sclerotherapy, radiofrequency ablation, and arterial stenting for arterial ulcers. However, for autoimmune pathologies like vasculitis ulcers and pyoderma gangrenosum, there is a notable paucity of standardized treatment practices or randomized controlled trials. These inflammatory atypical wounds often necessitate a more personalized approach based on clinical expertise and retrospective case reports, given the limited availability of established guidelines or evidence-based protocols for managing such conditions.

Our treatment approach offers several advantages. Firstly, it emphasizes cost-effectiveness by categorizing patients based on diagnostic findings, ensuring that the treatment does not impose an economic burden. Additionally, all patients receive outpatient care, minimizing hospitalization costs and disruptions to their daily lives. Notably, our approach appears to prevent serious side effects, such as hyperglycemia, possibly due to the anti-diabetic properties of colchicine. Furthermore, patients experience significant improvements in their quality of life, including pain relief, reduced edema, and enhanced pruritus control, often enabling them to return to work. Moreover, treatment responses to immunosuppressants aid in confirming autoimmune or inflammatory etiology. However, challenges include the absence of definite treatment recommendations for chronic ulcers and difficulties in patient compliance, often linked to the chronic nature of ulcers and the need for education on sterile wound care and dressing.

## Appendices

Supplementary table

**Table 2:** Patient characteristics and therapeutic regimen. Hcq: hydroxychloroquine; od: once daily; bd: twice daily. Case 21 - Discussed in detail as case report 1. Case 22 - Discussed in detail as case report 2. Case 23 - Discussed in detail as case report 3.

Case No.	Age	Gender	Diagnosis	Immune therapy	Time for wound healing (in months)
1	63	F	Atypical ulcer	Deflazacort 30 mg od, colchicine 0.5 mg od	1.25
2	60	F	Atypical ulcer	Deflazacort 30 mg od, colchicine 0.5 mg bd, hcq 200 mg od, dapsone 100 mg od, folate 5 mg od	3
3	56	F	Atypical ulcer	Deflazacort 30 mg od, colchicine 0.5 mg bd, hcq 200 mg bd, folate 5 mg od	3
4	62	M	Atypical ulcer	Deflazacort 30 mg od, colchicine 0.5 mg od, dapsone 100 mg od, folate 5 mg od	6
5	51	M	Atypical ulcer	Deflazacort 30 mg, colchicine 0.5 mg od, dapsone 100 mg od, folate 5 mg od, azathioprine 50 mg od	22
6	63	F	Venous ulcer	Deflazacort 6 mg bd, colchicine 0.5 mg bd, dapsone 100 mg od, folate 5mg od	1
7	83	M	Atypical ulcer	Deflazacort 6 mg bd, colchicine 0.5 mg od	2
8	41	M	Venous ulcer	Deflazacort 6 mg bd, colchicine 0.5 mg od	1
9	71	F	Atypical ulcer	Deflazacort 30 mg, colchicine 0.5 mg bd, hcq 200 mg od, dapsone 100 mg od, folate 5 mg od	7
10	37	F	Atypical ulcer	Deflazacort 6 mg, colchicine 0.5 mg od, dapsone 100 mg od, folate 5 mg od	1.5
11	58	M	Venous ulcer	Deflazacort 30 mg, colchicine 0.5 mg bd, dapsone 100 mg od, folate 5 mg od	7
12	67	F	Atypical ulcer	Deflazacort 6 mg od, colchicine 0.5 mg od, dapsone 100 mg od, folate 5 mg od	3
13	73	M	Atypical ulcer	Deflazacort 6 mg, colchicine 0.5 mg bd, hcq 200 mg od	5
14	54	F	Atypical ulcer	Deflazacort 30 mg od, colchicine 0.5 mg bd, dapsone 100 mg od, folate 5 mg od	7
15	55	M	Atypical ulcer	Deflazacort 6 mg od, colchicine 0.5 mg od, dapsone 100 mg od, folate 5 mg od, azathioprine 50 mg od	2
16	52	M	Diabetic neuropathy	Deflazacort 30 mg, colchicine 0.5 mg bd, dapsone 100 mg od, folate 5 mg od	6
17	62	F	Atypical ulcer	Deflazacort 30 mg od, colchicine 0.5 mg od	1
18	55	M	Venous ulcer	Deflazacort 30 mg od, colchicine 0.5 mg od, dapsone 100 mg od, folate 5 mg od	6
19	53	F	Atypical ulcer	Deflazacort 6 mg bd, colchicine 0.5 mg bd, dapsone 100 mg od	15
20	63	F	Atypical ulcer	Deflazacort 30 mg od, colchicine 0.5 mg bd, dapsone 100 mg od, azathioprine 50 od	6
21	72	F	Atypical ulcer	Deflazacort 30 mg od, colchicine 0.5 mg bd, dapsone 100 mg od, folate 5 mg od	5
22	63	M	Atypical ulcer	Deflazacort 30 mg od, colchicine 0.5 mg bd	12
23	54	M	Diabetic neuropathy	Deflazacort 6 mg bd, colchicine 0.5 mg bd, dapsone 100 mg	1
24	61	F	Atypical ulcer	Deflazacort 30 mg od, colchicine 0.5 mg bd, dapsone 100 mg od, folate 5 mg od	6
25	66	M	Atypical ulcer	Deflazacort 30 mg od, colchicine 0.5 mg bd, dapsone 100 mg od, folate 5 mg od	6
